# Long-term outcome of progressive multifocal leukoencephalopathy with recombinant interleukin-2 treatment and an associated increase in the number of HPyV-2-specific T-cells: a case report

**DOI:** 10.1177/20406207231201721

**Published:** 2023-10-09

**Authors:** Fieke W. Hoff, John Rolwes, Paula A. Hardeman, Molly Perkins, Eugene O. Major, Daniel Douek, Robert H. Collins, Benjamin M. Greenberg

**Affiliations:** Department of Internal Medicine, UT Southwestern Medical Center, Dallas, TX, USA; Harold C. Simmons Comprehensive Cancer Center, UT Southwestern Medical Center, Dallas, TX, USA; Department of Internal Medicine, UT Southwestern Medical Center, Dallas, TX, USA; Harold C. Simmons Comprehensive Cancer Center, UT Southwestern Medical Center, Dallas, TX, USA; Department of Neurology, UT Southwestern Medical Center, Dallas, TX, USA; Human Immunology Section, Vaccine Research Center, National Institute of Allergy and Infectious Diseases, National Institutes of Health, Bethesda, MD, USA; National Institute of Neurological Disorders and Stroke, National Institutes of Health, Bethesda, MD, USA; Human Immunology Section, Vaccine Research Center, National Institute of Allergy and Infectious Diseases, National Institutes of Health, Bethesda, MD, USA; Department of Internal Medicine, UT Southwestern Medical Center, Dallas, TX, USA; Harold C. Simmons Comprehensive Cancer Center, UT Southwestern Medical Center, 5323 Harry Hines Blvd, Dallas, TX 75390-8806, USA; Department of Neurology, O’Donnell Brain Institute, UT Southwestern Medical Center, 5323 Harry Hines Blvd, Dallas, TX 75390-8806, USA

**Keywords:** case report, follicular lymphoma, IL-2, non-Hodgkin lymphoma, progressive multifocal leukoencephalopathy

## Abstract

Progressive multifocal leukoencephalopathy (PML) is a demyelinating disease caused by reactivation of the human polyomavirus 2 (HPyV-2). PML is associated with a high morbidity and mortality rate and there is currently no standard curative therapy. We report short-term immunologic response and long-term clinical outcomes in a patient diagnosed with follicular lymphoma (FL) who developed PML. Diagnosis of PML was established conclusively based on findings from a brain biopsy. The patient was treated with recombinant interleukin 2 (IL-2) and showed rapid clinical improvement. HPyV-2-specific T-cells were tracked longitudinally and correlation with clinical status, viral load, and radiographic imaging was documented. After the progression of the patient’s FL, which required an allogeneic bone marrow transplant, the patient prophylactically received human leukocyte antigen-matched donor-derived HPyV-2 T-cells to prevent the recurrence of the PML as part of a clinical trial. Twelve years after the initial diagnosis of PML, he did not develop a relapse of his PML, supporting data that therapies that increase HPyV-2-specific T-cells, including IL-2, may be effective in the management of PML.

## Introduction

Progressive multifocal leukoencephalopathy (PML) is a rare relentlessly demyelinating disease caused by the reactivation of the human polyomavirus 2 (HPyV-2) (formerly known as the JC virus). HPyV-2 is a small non-enveloped polyomavirus with a circular double-stranded DNA genome that contains three main regions. The early region encodes for large T and small T antigens which are required for viral transformation, gene regulation, and replication, and are transcribed prior to DNA replication. Late genes are transcribed after gene transcription and encode for the agnoprotein and viral capsid proteins VP1, VP2, and VP3. The noncoding control regions contain transcription factor binding sites that regulate the expression of both early and late genes.^1,2^

Infection of the mucosal surfaces with HPyV-2 often happens asymptomatically and is common during childhood. Viremic spread occurs to other tissues and organs, and the infection is thought to persist quiescently in the host cell. In patients with compromised cellular immunity, however, HPyV-2 can undergo complex sequential DNA sequence alteration of the noncoding control region as well as in VP1, which may help explain the ability to, in rare cases, infect the oligodendrocytes of the central nervous system (CNS).^3,4^ Oligodendrocytes are the myelin-producing cells of the CNS, and destruction of the oligodendrocytes leads to demyelination that occurs in a multifocal distribution causing PML.^2,5,6^

PML is most commonly seen in patients with HIV/AIDS,^7^ as well as in patients with multiple sclerosis and Crohn’s disease who are treated with natalizumab, a monoclonal antibody against the alpha-4 subunit of integrin molecules on T cells, restricting trafficking of T cells to the CNS. Previously, PML was also seen in patients with psoriasis treated with efalizumab, an immunomodulatory monoclonal antibody against CD11a present in T cells.^8^ This drug was withdrawn from the market due to PML side effects.

PML is associated with a high morbidity and mortality rate.^9^ Neurological deficits are associated with the areas of demyelination of the brain. There is currently no standard therapy. The most effective strategy to treat PML is to reconstitute the immune response, and its success largely depends on early diagnosis and rapid and effective immune repletion. Symptoms significantly improved after treatment of HIV/AIDS patients with anti-retroviral therapy. In patients undergoing solid organ or bone marrow transplant, or with an auto-immune disorder, PML improved after reduction of immunosuppression.^10–13^ In patients where the compromised immune system cannot simply be reversed by discontinuation of immunosuppressive agents (e.g. in patients with a primary immunodeficiency or lymphoproliferative and myeloproliferative disorders), it has been reported that the immune response against the HPyV-2 can be improved by increasing the number and activity of HPyV-2-specific T-cells. This increase can be accomplished by either directly stimulating T cells with growth factors [e.g. interleukin (IL)-2, IL-7], or by adoptively transferring HPyV-2-specific T-cells.^14–17^ In addition, treatment with immune checkpoint inhibitors to facilitate the immune response has been investigated. While results were encouraging, checkpoint inhibition did not convincingly prolong survival or reduce neurological disability overall.^18,19^

In this report, we describe a patient diagnosed with advanced stage follicular lymphoma (FL) who developed PML in the setting of prior chemo-immunotherapy. Rapid and sustained improvement of his PML occurred after administration of recombinant IL-2, which was associated with a marked increase in HPyV-2-specific T-cells. During the course of his treatment, his FL progressed, requiring an allogeneic hematopoietic stem cell transplant (HSCT) from an unrelated donor. To prevent a flare-up of his prior PML, he prophylactically received donor-derived human leukocyte antigen (HLA)-matched T-cells with anti-HPyV-2 activity after his HSCT as part of a phase I clinical trial.^20^ He remained well 12 years after the initial diagnosis of PML, suggesting that therapies that increase HPyV-2-specific T-cells may be effective in the management of PML.

## Methods

Portions of cerebrospinal fluid and peripheral blood specimens were sent to the NIH for viral load calculations using quantitative PCR analysis and T-cell population analyses.^21^ As published previously, peripheral blood mononucleated cells were isolated and stimulated with five pools of peptides covering the HPyV-2 proteome: large T antigen, small T antigen, VP1, VP2, and agnoprotein.^22^ For measurement of cytomegalovirus (CMV)-specific T-cells, CMV pp65 peptides obtained from JPT Peptide Technology were used. Responses were measured by intracellular cytokine staining and polychromatic flow cytometry for cytokines associated with effective control of viral infections: interferon (IFN)-γ and tumor necrosis factor (TNF)-α. Given that expansion of memory T cells in the setting of PML occurs before naïve T cells^23^ and that low frequencies of central memory T cells are associated with a risk of PML,^24^ memory T-cell responses were measured. The reporting of this study conforms to the CARE case report guidelines.^25^

## Case

A 48-year-old male with no past medical history was diagnosed in May 2007 with FL Grade 2, Ann Arbor stage IV. The diagnosis was confirmed histologically on tissue obtained from a lymph node in the left inguinal area. He was treated with R-CHOP (rituximab, cyclophosphamide, doxorubicin, vincristine, and prednisone) for six cycles and attained complete remission. Subsequently, he received maintenance therapy with rituximab administered every 3 months for the first two cycles followed by every 6 months for the next cycles, until June 2009 when he relapsed. Because of the low-burden disease, he proceeded with watchful waiting. His disease further progressed in March 2010 and he was enrolled in an autologous recombinant idiotypic vaccine phase I clinical trial for the treatment of patients with relapsed or transformed FL (NCT01022255).^26^ He received three cycles of bendamustine resulting in a good clinical response. A fourth cycle of the bendamustine was deferred due to low blood counts. Twelve weeks after completion of the last dose of bendamustine, he received two subcutaneous injections of 1 mg anti-idiotype vaccine (Icon Genetics GmbH, Halle (Saale), Germany) each 4 weeks apart along with subcutaneous 125 µg granulocyte-macrophage colony-stimulating factor following the vaccination administration on Days 1–4.

**Figure 1.**
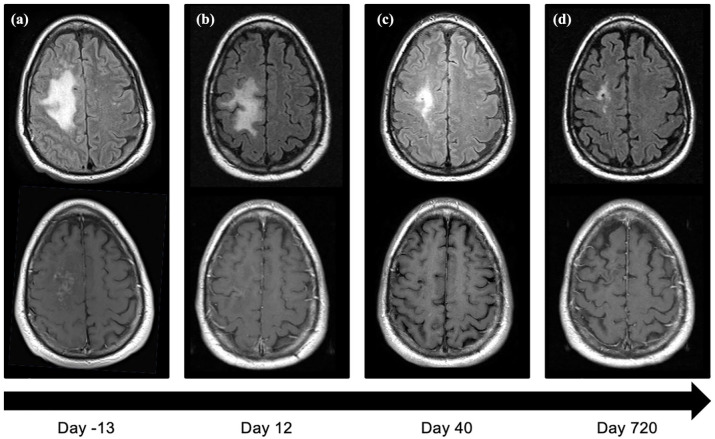
(a) Imaging showing the initial axial fluid-attenuated inversion recovery magnetic resonance imaging (MRI) of the patient’s brain, showing a hyper-intensive lesion in the posterior right frontal lobe. Typical for PML is sparing of the more superficial cortex. (b–d) Images obtained at days 12, 40, and 720 post-treatment initiation, showed significant resolution based on a decrease in the hyperintensity of the lesion. PML, progressive multifocal leukoencephalopathy.

**Figure 2.**
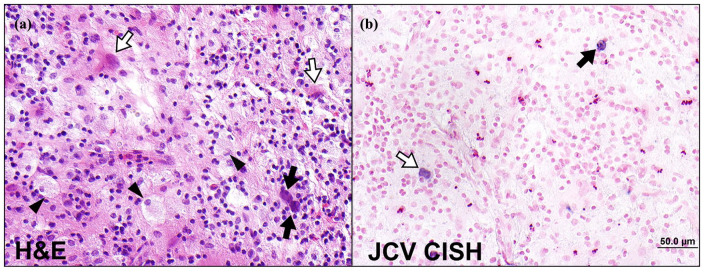
Histopathology of brain biopsy. (a) H&E stain showing enlarged astrocytes with a large inclusion-bearing nucleus, (b) chromogenic *in situ* hybridization (CISH) for the JC virus. H&E, hematoxylin and eosin.

Two weeks after administration of the idiotypic vaccine, however, he developed marked weakness and a pronator drift of his left upper extremity and confusion. A magnetic resonance imaging (MRI) of the brain was performed, showing a lesion in the posterior right frontal lobe with mass effect and patchy enhancement [Figure 1(a)]. While awaiting the final diagnosis and to reduce the inflammation, he was empirically treated with dexamethasone 4 mg twice daily for a total of 10 days. Stereotactic needle biopsy of the brain lesion demonstrated inflammatory infiltrates composed of CD68^+^ macrophages, reactive astrocytes, and CD3^+^ small lymphocytes, as well as several, enlarged, highly hyperchromatic nuclei positive for HPyV-2 by *in situ* hybridization consistent with a diagnosis of PML (Figure 2). Participation in the idiotype vaccine trial was discontinued.

To increase HPyV-2-specific T-cells to treat the PML, he was then treated with recombinant IL-2; 500,000 units of IL-2 subcutaneously on Day 1 followed by 1,000,000 units daily thereafter. Dexamethasone 4 mg twice daily was continued for an additional 7 days as prophylaxis for immune reconstitution inflammatory syndrome. He reported subjective clinical improvement within a few days, and after 1 week his physical examination was significantly improved in left upper extremity strength. An MRI performed on Day 12 showed evident resolution of previous radiographic abnormalities [Figure 1(b)–(d)]. He continued to improve clinically and radiographically and returned to full daily activities. He remained on IL-2 therapy continuously.

**Figure 3.**
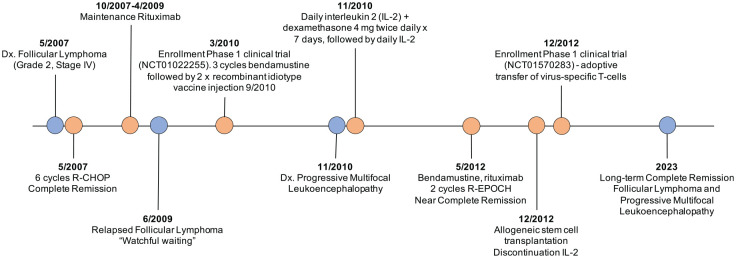
Timeline of his diagnostic and treatment history.

His FL gradually progressed and by May 2012 he had extensive disease requiring further therapy. He received one additional cycle of bendamustine and rituximab, radiotherapy, and two cycles of R-EPOCH (rituximab, etoposide, prednisone, vincristine, cyclophosphamide, doxorubicin). A near-complete response to therapy was achieved and in December 2012, he underwent allogeneic HSCT from an HLA-matched unrelated donor utilizing a reduced intensity preparative regimen with fludarabine and melphalan. IL-2 treatment was discontinued 1 week before the stem cell infusion date. Post-HSCT immunosuppression included treatment with tacrolimus and methotrexate. Given his history of PML and risk of reactivation following HSCT, he received a single prophylactic infusion of HLA-matched donor-derived polyomavirus reactive T cells (5 × 10^6^ cells/m^2^ body surface) on Day +41 post-HSCT as part of the phase I clinical trial (NCT01570283). Virus-specific cytotoxic T-cells were made by direct stimulation of donor-derived peripheral blood mononuclear cells with overlapping peptide libraries that incorporated antigens from the Epstein–Barr virus, CMV, adenovirus, human herpes virus, and BK virus (with cross-recognition of the HPyV-2 homolog sequence).^20^ More information about the generation of the T-cell-specific cells is published by Papadopoulou *et al.*^20^ His allogeneic HSCT was complicated by mild chronic graft-*versus*-host disease (GVHD) involving the skin and liver that responded to tacrolimus and corticosteroids. Corticosteroids were tapered off and he remained on a small dose of tacrolimus. He did not develop any opportunistic infections. Twelve years after his PML diagnosis, there was no evidence of recurrence of the PML, and his lymphoma remained in remission. A timeline highlighting his disease and treatment history is illustrated in Figure 3.

## Results

**Figure 4.**
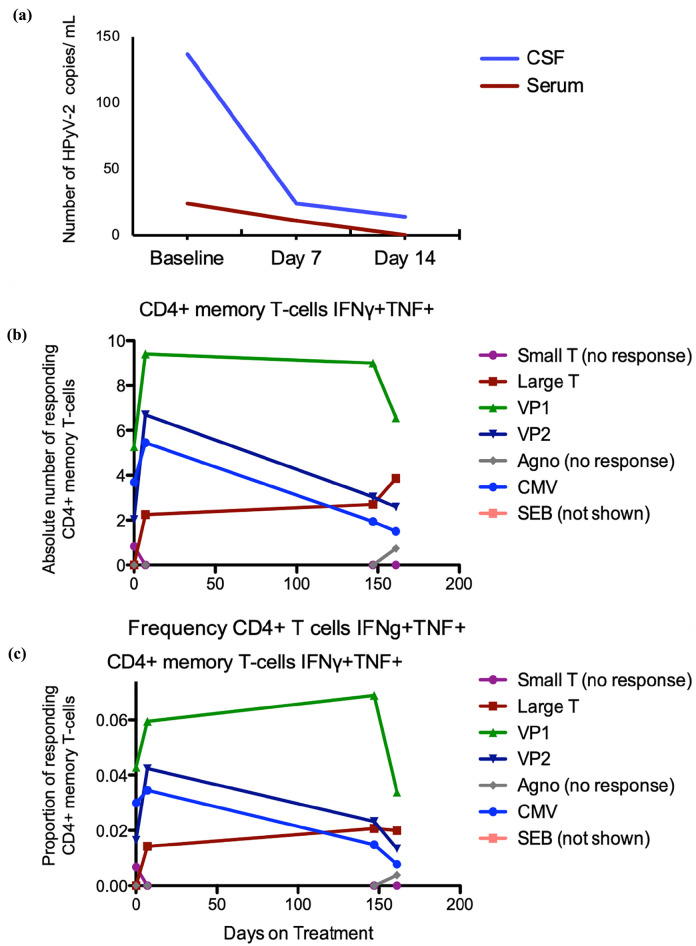
(a) Decrease in a number of HPyV-2 copies in cerebrospinal fluid and serum after the start of interleukin 2 treatment. (b, c) Absolute number and frequency as a proportion to the general T-cell population of CD4^+^ memory T cells by IFN-γ and TNF-α production after stimulation with peptides covering the HPyV-2 proteome: large T antigen, small T antigen (early regulatory proteins), VP1, VP2 (major and minor viral capsid proteins), and agnoprotein (late regulatory protein), as well as a cytomegalovirus antigen and mitogen staphylococcal enterotoxin B (SEB). HPyV-2, human polyomavirus 2; IFN, interferon; TNF, tumor necrosis factor; VP, viral capsid protein.

During the treatment with recombinant IL-2, there was a clinical and radiographic response (Figure 1) and normalization of his neurologic status. Biochemical analysis of his blood and CSF specimens showed a reduction in viral load [Figure 4(a)] that occurred during the expansion of HPyV-2-specific T-cell populations [Figure 4(b) and (c)]. The absolute number and frequency of CD4^+^ T cells by IFN-γ and TNF-α production were measured after stimulation with peptides covering the HPyV-2 proteome, including the large T antigen and small T antigen, both early regulatory proteins, VP1 and VP2, a major and minor viral capsid protein, and the agnoprotein, which is a late regulatory protein. After the start of treatment with IL-2, an increase was seen in HPyV-2-specific T-cells directed against the large T, VP1, and VP2, as well as in T cells responding to the CMV. No increase was seen in T cells directed against the small T antigen, the agnoprotein, or mitogen staphylococcal enterotoxin B.

## Discussion

Primary infection with HPyV-2 is generally asymptomatic and most adults are infected with HPyV-2 in a persistent latent state.^27^ Although the mechanisms of viral control are not completely delineated, both humoral and cellular mechanisms, in particular virus-specific CD4^+^ and CD8^+^ T-cells, are understood to play a role.^28^ States of immunosuppression can allow the virus to proliferate and infect oligodendrocytes leading to PML.^9^ Our patient had previously received rituximab and bendamustine, both of which have been associated with PML.^29–33^ The prognosis of PML is very poor but good outcomes have been seen in patients who received treatments aimed at increasing T-cell numbers and function. We had previously observed improved CD4^+^ T-cell numbers and resolution of opportunistic infections in a patient with idiopathic CD4 lymphopenia treated with IL-2,^34^ and we reasoned that our patient who was immunocompromised from chemotherapy and immunotherapy might benefit from recombinant IL-2 treatment. Our patient had significant clinical and radiographic improvement soon after beginning IL-2 therapy. This clinical improvement was associated with an expansion of HPyV-2 CD4^+^ memory T cells (Figure 4). The patient remains free of PML relapse several years after therapy.

A few additional points are worth addressing with regard to this patient’s particular case. First, the patient developed symptoms fairly soon after receiving an idiotype vaccine, raising the question of whether the vaccine may have caused or unmasked the PML. While this is possible, a mechanistic explanation is unclear and PML was not observed in other patients in this trial (*n* = 27)^26^ or in other anti-idiotype vaccine trials. Second, it is worth noting that we administered IL-2 for a prolonged period after the patient’s initial response. This was done because of concern for high risk of PML relapse, in view of the patient’s state of ongoing immunosuppression from his lymphoma and prior immunosuppressive therapy. Whether the prolonged IL-2 may have had any role in the patient’s subsequent lymphoma relapse is speculative; we would note that the patient’s risk of lymphoma relapse was quite high on clinical grounds, in view of his aggressive initial presentation and, in particular, his short disease-free interval after initial therapy. Lastly, this patient received virus-specific T-cells from his allogeneic HSCT donor as part of a clinical trial.^20^ At the time of his allogeneic HSCT, the patient did not have evidence of PML. However, we were concerned about the possibility of PML relapse in the setting of profound post-allogeneic HSCT-associated immunosuppression. We were reluctant to continue IL-2 in the post-transplant setting because of the possibility of exacerbating GVHD. Therefore, we referred the patient for participation in a clinical trial investigating infusion of allogeneic donor-derived virus-specific T-cells which included T cells with activity against BK virus antigens (which cross-react with HPyV-2 antigens). The patient did not have recurrence of PML but the role of the allogeneic virus-specific T-cells is unknown since the patient had no evidence of disease when the cells were given.

Only a handful of cases have been described showing clinical improvement in PML patients when treated with IL-2.^14,35–38^ Our results add to these previous observations by demonstrating a marked rise in HPyV-2-specific T-cells in association with resolution of HPyV-2 viremia and clinical improvement, which was not measured in the majority of prior reports. Case reports have also described varied clinical improvement after treatment with other T-cell-stimulating ILs, including IL-7,^15–17,39–42^ a cytokine regulating peripheral T-cell survival and homeostasis, and the IL-15 super-agonist N-803,^43^ which is critical for the proliferation and activation of natural killer cells and CD8^+^ memory T cells. A retrospective observational study of 64 patients reported a 1-year overall survival following recombinant IL-7 initiation of only 54%.^44^ Based on the literature, there are no associations described between the development of lymphoma and IL treatment. Given that the patient had demonstrated the development of a clinically aggressive FL with early relapse that occurred prior to the onset of the PML, we believe that the recurrence of his lymphoma was unlikely associated with prolonged IL-2 therapy.

Other approaches that have been studied to improving the immune control of the HPyV-2 aiming to increase in HPyV-2-specific CD4^+^ and CD8^+^ T cells include viral-like particle vaccines,^42,45–50^ immune checkpoint inhibitors, and adoptive transfer of polyclonal T cells. The immune checkpoint inhibitors pembrolizumab and nivolumab have been investigated based on the observation that the expression of programmed cell death protein 1 is elevated in blood and CSF CD4^+^ and CD8^+^ T cells in patients with PML.^18,19,51–53^ Mixed results have been reported. The largest retrospective study that included 79 PML patients reported a 1-year survival of 51%.^54^

Partially HLA-matched multi-virus-specific polyclonal CD4^+^ and CD8^+^ cells with or without *ex vivo* expansion have shown promise in treating active infections in patients with post-solid organ transplantation or HSCT or with other immunosuppressed states but have only been described in a small number of cases.^20,55–58^ Papadopoulou *et al.*^20^ reported eleven patients of which eight patients had up to four active infections, with a virological and clinical response in 94%. Another study reported three patients with PML treated with virus-specific T-cells with *ex vivo* expansion of which two showed clinical improvement and the third had stabilization of the disease with a reduction in the HPyV-2 viral load.^57^

In summary, PML that has occurred in the setting of chemotherapy or immunotherapy-associated immunosuppression is considered to have a dismal prognosis. Our observation and others suggest that the disease in this setting might be amenable to treatment with approaches that increase the numbers of HPyV-2-specific T-cells. Further investigation with controlled studies as well as evaluation of immunological correlations is encouraged.

## Declarations

**Ethics approval and consent to participate:** The patient provided written consent for the off-label use of IL-2 in the treatment of his PML and to the IRB-approved UT Southwestern Medical Center (Dallas, TX, USA) Neuroscience’s biorepository protocol allowing for specimen storage and data collection during the course of his care. The use of IL-2 was off-label for this indication and not used in a clinical investigation, IRB or ethics approval is therefore not required. The patient voluntarily provided written informed consent for publication of their medical data and images.

**Consent for publication:** Not applicable.

**Availability of data and materials:** Not applicable.
